# Semi-SwinUNeTR: Towards 3D Swin Vision Transformer-Based UNet for Medical Image Segmentation with Limited Annotations

**DOI:** 10.3390/bioengineering13060695

**Published:** 2026-06-17

**Authors:** Yinbing Tian, Ziyang Wang, Li Guo

**Affiliations:** 1 School of Artificial Intelligence, Beijing University of Posts and Telecommunications, Beijing 100876, China; 2 Engineering Research Center of Blockchain and Network Convergence Technology, Ministry of Education, Beijing University of Posts and Telecommunications, Beijing 100876, China; 3 National Engineering Research Center for Mobile Internet Security Technology, Beijing University of Posts and Telecommunications, Beijing 100876, China; 4 Key Laboratory of Universal Wireless Communications, Ministry of Education, Beijing University of Posts and Telecommunications, Beijing 100876, China; 5 School of Computer Science and Digital Technologies, Aston University, Birmingham B4 7ET, UK

**Keywords:** volumetric medical image segmentation, vision transformer, semi-supervised learning, SwinUNeTR, auxiliary weighting, interpolation consistency, biomedical image analysis

## Abstract

Accurate brain tumor segmentation from magnetic resonance imaging (MRI) is essential for computer-assisted diagnosis, treatment planning, and disease monitoring. However, brain tumors usually exhibit irregular, heterogeneous, and multi-scale spatial patterns with complex and ambiguous boundaries. At the same time, the performance of deep segmentation models is often constrained by the limited availability of voxel-level annotations, which are expensive and time-consuming to obtain. To address these challenges, this paper proposes Semi-SwinUNeTR, a semi-supervised framework for 3D brain tumor segmentation with limited annotated data. The proposed method adopts SwinUNeTR as the segmentation backbone, enabling hierarchical volumetric representation learning through shifted-window self-attention while preserving the encoder–decoder structure required for dense prediction. On top of this backbone, we introduce a dual-consistency semi-supervised learning strategy, consisting of mean teacher-based model consistency and interpolation consistency-based data consistency. In addition, voxel-wise consistency weights are used to redistribute semi-supervised supervision toward structurally complex and boundary-irregular tumor regions without changing the SwinUNeTR backbone. Experiments on the BraTS 2019 benchmark demonstrate that the proposed framework achieves strong performance across different annotation ratios. The original Semi-SwinUNeTR achieves Dice scores of 84.93%, 86.25%, 87.05%, and 87.83% under the 10%, 20%, 40%, and 80% labeled-data settings, respectively. With the weighted consistency extension, the Dice scores are further improved to 85.64%, 87.94%, and 88.59% under the 10%, 20%, and 80% labeled-data settings, respectively, while the corresponding HD_95_ values are reduced to 8.9826, 8.1854, and 7.4533. These results indicate that combining a SwinUNeTR backbone with complementary model consistency, data consistency, and voxel-wise consistency weighting is an effective strategy for semi-supervised volumetric medical image segmentation under limited annotation.

## 1. Introduction

Medical image segmentation stands as a cornerstone technique in the realm of medical imaging analysis. At its core, segmentation involves the partitioning of digital medical images into multiple segments, often aiming to identify and delineate specific anatomical structures, pathological abnormalities, or other regions of interest [1,2,3,4,5,6]. This process is paramount in transforming raw imaging data into a structured format that can be readily interpreted and analyzed. In clinical settings, accurate segmentation is instrumental in tasks ranging from diagnosis and treatment planning to tracking disease progression and response to therapy [7,8]. Given the intrinsic variability of human anatomy and the nuances of diseases, automated and accurate segmentation becomes pivotal. It not only reduces the reliance on manual and time-consuming delineations by experts but also ensures a consistent and objective evaluation, thereby paving the way for improved patient care and advanced medical research [9,10,11,12].

From a geometric perspective, brain tumors often present irregular shapes, heterogeneous textures, and multi-scale spatial patterns. Their boundaries are rarely smooth or simply connected, and may instead exhibit complex geometry caused by infiltrative growth and heterogeneous tissue appearance. This makes brain tumor segmentation not only a dense prediction problem, but also a challenging task of learning complex non-Euclidean and multi-scale anatomical structures from volumetric images. Therefore, representation learning methods that can capture both local boundary details and long-range contextual dependencies are particularly important for robust segmentation.

One of the pioneering architectures in this realm is the U-Net [1]. Designed specifically for biomedical image segmentation, U-Net is characterized by its symmetric encoding and decoding paths, which are connected by skip connections. This design facilitates the capture of both high-level features and fine-grained details, making it particularly adept at handling the intricacies of medical images. Following the success of U-Net [1], several variants and improvements were proposed. U-Net++ and U-Net3+ further enhanced the original design by introducing nested and dense skip pathways, enabling even finer multi-scale feature extraction [13,14]. V-Net, tailored for volumetric medical images, extended the 2D U-Net paradigm into 3D, catering to the depth dimension inherent in many medical imaging modalities [15]. ResU-Net incorporated residual connections, improving gradient flow and allowing for deeper architectures [5]. DenseU-Net amalgamated the dense connections from DenseNet into the U-Net framework, enhancing feature reuse [16,17,18]. Meanwhile, Attention U-Net integrated attention mechanisms, enabling the model to focus on more relevant regions during segmentation. However, the deep learning landscape witnessed a paradigm shift with the introduction of Transformers, initially designed for natural language processing tasks [19]. When adapted to the visual domain, the Transformer architecture, which leverages self-attention mechanisms, allowed for long-range dependencies and dynamic receptive fields in images. Vision Transformer (ViT) segmented images into fixed-size patches and processed them as a sequence, proving competitive with traditional CNNs [20,21]. Swin Transformer furthered this approach, introducing hierarchical structures and local patches for scalability. In the realm of segmentation, architectures like SegFormer integrated Transformers with familiar segmentation paradigms for enhanced results [22]. Swin U-Net and TransUNet are exemplary hybrid models, merging the traditional U-Net architecture with Transformer-based features, providing both the spatial precision of U-Nets and the global context-awareness of Transformers. These developments underscore the potential and adaptability of deep learning models, revealing the expansive horizon in the quest for impeccable medical image segmentation.

To effectively address the challenge of limited annotated data, which are often expensive and time-consuming to obtain, semi-supervised learning has emerged as an important paradigm in medical image segmentation. Building on the progress of U-Net-based architectures [1,23,24] and the paradigm shift introduced by Transformer models [19,22,25], semi-supervised segmentation methods aim to exploit both labeled and unlabeled data to improve accuracy and generalization. Among existing strategies, consistency regularization has become one of the most widely used principles. Its central assumption is that a segmentation model should produce stable and semantically consistent predictions when the same image is subjected to reasonable perturbations. These perturbations can be introduced at different levels. At the data level, they may include image augmentation, intensity transformation, noise injection, spatial transformation, or sample interpolation. At the model level, they may arise from different network initializations, dropout, parameter perturbation, or teacher–student models with temporally averaged weights [26]. By enforcing prediction consistency under such perturbations, semi-supervised models can extract additional supervision from unlabeled data without requiring manual annotations. Another common strategy is pseudo-labeling, where confident predictions on unlabeled images are used as auxiliary training targets [27,28,29]. These techniques, particularly when combined with strong segmentation backbones such as U-Net and Vision Transformer variants, provide an effective route to reduce the dependence on dense annotations and develop more reliable medical image segmentation systems.

Drawing on the aforementioned advancements and recognizing the inherent strengths and limitations of existing models, this paper introduces a robust semi-supervised segmentation network that combines multiple distinct models for medical imaging. Therefore, the proposed method differs from existing teacher–student approaches by explicitly coupling volumetric Transformer representation, complementary model- and data-level consistency learning, and structurally aware supervision for complex tumor morphology. Our contributions are manifold:

1. We propose a semi-supervised 3D medical image segmentation framework based on SwinUNeTR, which combines the hierarchical representation ability of shifted-window self-attention with the encoder–decoder structure required for dense volumetric prediction.
2. We introduce an interpolation consistency-based data consistency strategy for unlabeled MRI volumes. By mixing unlabeled images and their corresponding soft pseudo-labels, the student network is encouraged to produce smooth and consistent predictions in the interpolated data space.
3. We incorporate a Mean Teacher-based model consistency mechanism, where the teacher model is updated by the exponential moving average of the student parameters. This design provides more stable pseudo-supervision for unlabeled samples and reduces the influence of noisy predictions during training.
4. We introduce an auxiliary structure-weighted consistency strategy, where a local complexity map is incorporated into the consistency objective only as a voxel-wise weighting parameter. By redistributing consistency supervision toward structurally complex regions, the proposed strategy improves the modelling of irregular and heterogeneous tumor boundaries.
5. We conduct extensive experiments on the BraTS2019 dataset under different labeled-data ratios. The results demonstrate that the proposed Semi-SwinUNeTR consistently improves the SwinUNeTR backbone with the proposed dual-consistency training strategy and its auxiliary weighting parameter, especially for irregular and boundary-complex tumor regions under limited annotation.

## 2. Related Work

### 2.1. Medical Image Segmentation

The traditional medical image segmentation without machine learning mainly uses thresholds, seed growing or other process-based methods. However, they were unable to solve complicated tasks and still requires manual selection and adjustment. Early attempt on medical image segmentation methods were mainly contour-based and used traditional machine learning algorithms, as demonstrated by Tsai and others [30]. With the development of deep convolutional neural networks (CNNs), Ronneberger proposed U-Net for medical image segmentation [1]. Due to the simplicity and superior performance of the U-shaped structure, various U-Net-like methods have emerged, such as Weighted Res-UNet by Xiao [31], H-DenseUNet by Li [32], UNet++ by Zhou [23], and UNet 3+ by Huang [24]. This approach has also been introduced into the field of 3D medical image segmentation, as seen in the work of Çiçek in 2016 with 3D U-Net and with V-Net [15]. Currently, CNN-based methods have achieved tremendous success in medical image segmentation due to their powerful representation ability, particularly for tasks like tumor delineation and organ segmentation. These efforts aim to bridge the gap between the availability of diverse medical image datasets and the practicality of training models that can seamlessly adapt to various clinical scenarios, a crucial aspect in developing effective tools for healthcare applications.

### 2.2. Vision Transformer

The Transformer was first proposed for machine translation by Vaswani [19]. In the natural language processing (NLP) domain, Transformer-based methods, including the BERT model by Devlin [33], have achieved state-of-the-art performance in various tasks. Driven by this success, Dosovitskiy [25] introduced the Vision Transformer (ViT), achieving an impressive speed-accuracy trade-off on image recognition tasks. ViT’s drawback is its requirement for pre-training on a large dataset, which Touvron [34] addressed with training strategies in their Data-efficient image transformers (DeiT) work. Recently, notable works based on ViT by Wang [35], Han [36] and Liu [22] has been proposed: The Swin Transformer, an efficient hierarchical vision Transformer using a shifted windows mechanism, achieved state-of-the-art performance in tasks like image classification, object detection, and segmentation. ViTs have gained popularity for capturing long-range dependencies in images using self-attention mechanisms, offering advantages like improved transferability across domains and data modalities. It has been proven to be proficient in image classification and extend to tasks such as object detection, segmentation, and video analysis [1,19,37,38,39].

### 2.3. Semi-Supervised Segmentation

Semi-supervised segmentation is a key approach to overcoming the lack of annotated medical imaging data by using both labeled and unlabeled data to improve segmentation models. Recent advancements include methods like consistency regularization, self-training, and adversarial learning. Consistency regularization, highlighted by Lecouat [40], ensures models produce similar outputs for perturbed inputs, enhancing robustness. Han suggest that consistency-regularization with mixup might promote causality invariance [41]. Self-training uses the model’s predictions on unlabeled data as pseudo-labels to refine segmentation. Adversarial learning, used by Radford and Dumoulin [42,43], employs GANs to generate realistic image variations, enriching training with synthetic data. Goodfellow used the discriminator from a GAN to extract features for supervised models [44]. These methods have significantly improved segmentation accuracy and generalization, especially when large annotated datasets are unavailable. Incorporating these semi-supervised techniques into medical image segmentation frameworks promises to develop more reliable diagnostic tools, optimizing resource use and advancing data-constrained fields in medical imaging [45,46,47,48]. Although these methods have shown strong potential, most existing teacher–student and consistency-based segmentation approaches mainly enforce uniform prediction consistency over the whole image or volume. Such a design may not fully account for the spatially heterogeneous difficulty of brain tumor segmentation, where irregular boundaries and complex tumor textures can be more challenging than homogeneous background regions. In contrast, the proposed framework combines interpolation consistency and mean teacher consistency within a 3D SwinUNeTR backbone, and further uses an auxiliary voxel-wise weighting parameter to make unlabeled consistency regularization more sensitive to local structural complexity.

### 2.4. Auxiliary Structural Analysis in Biomedical Images

In addition to conventional convolutional and Transformer-based segmentation models, geometric descriptors have also been explored in biomedical image analysis [49,50]. Such descriptors can summarize irregular shape, boundary complexity, and texture heterogeneity, while related mathematical modelling can describe non-locality and memory effects in complex systems. These properties are relevant to brain tumor segmentation because tumor regions often show heterogeneous appearance and irregular contours across multiple spatial scales.

Recent studies have explored structure-guided learning for semi-supervised medical segmentation and related geometric analysis in foundation-model-based medical image segmentation [49,50]. In this work, the local structural descriptor is not treated as a separate model component. Instead, it is used only as an auxiliary weighting parameter within the same interpolation operator applied to unlabeled images and teacher-generated soft pseudo-labels.

## 3. Approach

In the task of 3D MRI semantic segmentation, the training data consist of two parts: the labeled data $D_{l}=\left\{\left(x_{i},y_{i}\right)\;|\;i=1,\ldots ,N_{l}\right\}$ and the unlabeled data $D_{u}=\left\{u_{j}\;|\;j=1,\ldots ,N_{u}\right\}$, where $x_{i}$ and $u_{j}$ denote input 3D MRI volumes, $y_{i}$ denotes the corresponding voxel-wise annotation for the labeled sample $x_{i}$, and $N_{l}$ and $N_{u}$ denote the numbers of labeled and unlabeled images, respectively. Given an input volume *x*, the segmentation network produces a voxel-level prediction $\hat{y}=f\left(x\right)$, where f(·) denotes a generic mapping from the input image to the predicted segmentation output, and $\hat{y}$ denotes the corresponding predicted mask or probability map. For labeled samples, the prediction ${\hat{y}}_{i}=f\left(x_{i}\right)$ can be directly compared with the reference annotation $y_{i}$. For unlabeled samples, the corresponding predictions are used to provide auxiliary supervision during training. In the final evaluation, the performance of the trained model is assessed by comparing the predicted segmentation against the ground truth on the test set.

**Figure 1:**
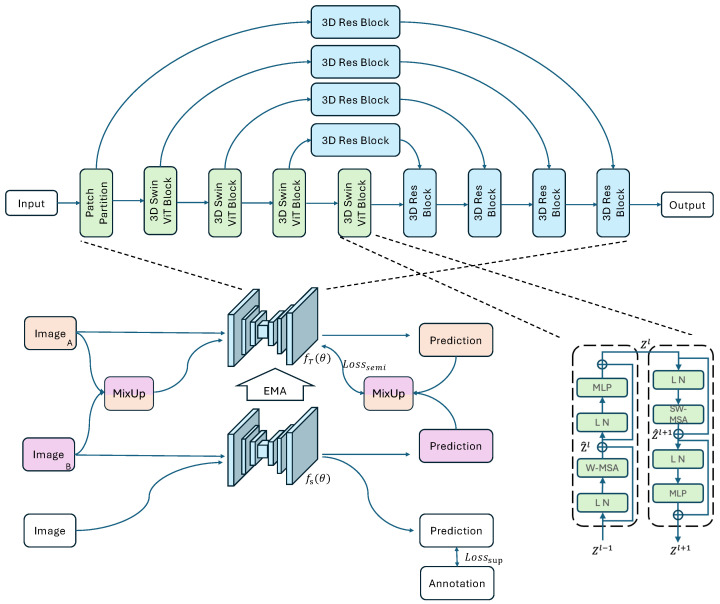
The Framework of 3D Semi-Supervised Swin Vision Transformer-Based UNet for Medical Image Segmentation.

The architecture of Semi-SwinUNeTR is briefly illustrated in Figure 1. The proposed framework consists of two 3D Swin ViT-based networks with the same architecture, namely a teacher network $f_{t}\left(\cdot ;\bar{\theta }\right)$ and a student network $f_{s}\left(\cdot ;\theta \right)$, where $\bar{\theta }$ and θ denote the parameters of the teacher and student models, respectively. For a given input *x*, the corresponding outputs of the two branches can be written as ${\hat{y}}_{t}=f_{t}\left(x;\bar{\theta }\right)$ and ${\hat{y}}_{s}=f_{s}\left(x;\theta \right)$. During training, the student branch is optimized as the main learning network, while the teacher branch serves as a more stable guidance branch. In the overall framework, the student is fed with strongly augmented data and the teacher is fed with weakly augmented data. The detailed designs of the network backbone, data consistency, model consistency, and auxiliary consistency weighting will be introduced in the following subsections.

From the optimization perspective, the proposed framework is trained by combining a supervised loss and an auxiliary weighted semi-supervised loss. The supervised loss is denoted by $L_{\sup}$ and is computed on the labeled subset $D_{l}$, measuring the discrepancy between the student prediction and the ground-truth annotation, i.e., between $f_{s}\left(x_{i};\theta \right)$ and $y_{i}$. The auxiliary weighted semi-supervised loss is denoted by $L_{\mathrm{semi}}^{\mathrm{aw}}$ and is computed mainly on the unlabeled subset $D_{u}$. It encourages the model to learn consistent predictions from unlabeled samples under the teacher–student training scheme, while using a local structural descriptor only as a weighting parameter for structurally complex tumor regions. Accordingly, the overall objective function can be written in the general form

(1) $$L=L_{\sup}+\lambda_{u}\left(t\right)L_{\mathrm{semi}}^{\mathrm{aw}},$$

where $\lambda_{u}\left(t\right)$ is a time-dependent weighting coefficient used to balance the contribution of the auxiliary semi-supervised term during training. At this stage, this formulation serves to establish the notation and the overall learning objective of the proposed framework. The specific forms of $L_{\sup}$ and $L_{\mathrm{semi}}^{\mathrm{aw}}$, together with the detailed mechanisms used to construct semi-supervised supervision signals, are described in the subsequent subsections.

### 3.1. Towards a Vision Transformer-Based U-Shape Network for Volumetric Segmentation

SwinUNeTR is developed as the backbone is motivated by the requirements of volumetric medical image segmentation [51]. First, brain tumor segmentation requires the model to capture long-range anatomical and pathological dependencies across the whole MRI volume, for which Transformer-based representations are more suitable than purely local convolutional operations. Second, the task is inherently three-dimensional, since tumor appearance, spatial extent, and boundary continuity are distributed across adjacent slices rather than isolated 2D images. Third, directly applying global self-attention to 3D volumes is computationally expensive. SwinUNeTR provides a practical solution by combining shifted-window self-attention with a hierarchical U-shaped encoder–decoder design, allowing the network to model volumetric context efficiently while preserving high-resolution spatial details for dense prediction. This design is also relevant to the analysis of irregular biomedical structures with complex spatial characteristics. Instead of relying on handcrafted geometric descriptors as the main representation, the hierarchical Swin Transformer encoder learns multi-scale representations directly from volumetric data, while shifted-window attention enables information exchange across local and neighbouring regions. This allows the network to better capture both fine boundary variations and broader anatomical context.

Both the teacher and student branches adopt the same segmentation backbone, namely a Vision Transformer-based U-shape network for 3D volumetric segmentation. Given an input MRI volume $X\in R^{H\times W\times D\times 4}$, where *H*, *W*, and *D* denote the spatial dimensions and 4 denotes the number of input modalities, the network learns a voxel-wise mapping

(2) $$\hat{Y}=f\left(X\right),$$

where f(·) denotes the segmentation network and $\hat{Y}$ denotes the predicted segmentation map. For the binary segmentation setting considered in this work, $\hat{Y}\in {\left[0,1\right]}^{H\times W\times D}$.

The overall architecture follows a U-shaped encoder–decoder design, in which a hierarchical 3D Swin Transformer encoder is used to capture long-range contextual dependencies, while a residual convolutional decoder is used to progressively recover spatial details. The input volume is first partitioned into non-overlapping 3D patches and projected into an embedded token representation. After patch partition, the shallow feature can be written as

(3) $$E_{0}\in R^{\frac{H}{2}\times \frac{W}{2}\times \frac{D}{2}\times C},$$

where *C* denotes the embedding dimension. The encoder then extracts a hierarchy of multi-scale feature maps through successive 3D Swin ViT blocks. Accordingly, the feature resolutions are progressively transformed as

(4) $$E_{1}\in R^{\frac{H}{4}\times \frac{W}{4}\times \frac{D}{4}\times 2C},\;E_{2}\in R^{\frac{H}{8}\times \frac{W}{8}\times \frac{D}{8}\times 4C},\;E_{3}\in R^{\frac{H}{16}\times \frac{W}{16}\times \frac{D}{16}\times 8C}.$$

At the deepest level, the bottleneck representation is obtained after the final hierarchical stage, providing the most compact and semantically rich feature representation. In this way, the encoder gradually reduces spatial resolution while increasing channel dimensionality, thereby constructing a coarse-to-fine representation hierarchy for volumetric segmentation.

Each 3D Swin ViT block contains two consecutive Transformer layers based on window self-attention. Specifically, a window-based multi-head self-attention (W-MSA) layer and a shifted-window multi-head self-attention (SW-MSA) layer are alternately employed to model both local and cross-window interactions. Their outputs can be written as

(5) $$\begin{array}{cc} {\hat{z}}^{l} & =W\text{-}\mathrm{MSA}\left(\mathrm{LN}\left(z^{l-1}\right)\right)+z^{l-1},\\ z^{l} & =\mathrm{MLP}\left(\mathrm{LN}\left({\hat{z}}^{l}\right)\right)+{\hat{z}}^{l},\\ {\hat{z}}^{l+1} & =\mathrm{SW}\text{-}\mathrm{MSA}\left(\mathrm{LN}\left(z^{l}\right)\right)+z^{l},\\ z^{l+1} & =\mathrm{MLP}\left(\mathrm{LN}\left({\hat{z}}^{l+1}\right)\right)+{\hat{z}}^{l+1}, \end{array}$$

where $z^{l}$ and ${\hat{z}}^{l}$ denote intermediate token features at layer *l*, LN denotes layer normalization, and MLP denotes a multi-layer perceptron. The self-attention operation is defined as

(6) $$\mathrm{Attention}\left(Q,K,V\right)=\mathrm{Softmax}\left(\frac{QK^{T}}{\sqrt{d}}+B\right)V,$$

where *Q*, *K*, and *V* denote the query, key, and value matrices, respectively, *d* denotes the feature dimension of the query and key, and *B* denotes the relative position bias. In the W-MSA layer, attention is computed within fixed M×M×M local windows. In the SW-MSA layer, the windows are shifted by half the window size, allowing information exchange across neighbouring windows and improving cross-region modelling.

Following the Transformer layers, patch merging is applied to aggregate neighbouring 2×2×2 tokens and project them to a new feature space with reduced spatial resolution and increased channel dimension. This hierarchical design enables the network to balance computational efficiency and representation capacity, which is particularly important for 3D medical images with large volumetric input size.

The decoder mirrors the encoder in a progressive upsampling manner and reconstructs high-resolution segmentation details from the multi-scale encoder representations. At each decoding stage, the feature is first refined by a 3D residual block consisting of two 3×3×3 convolutional layers with normalization. The refined feature is then upsampled by a deconvolutional layer, concatenated with the corresponding encoder feature through a skip connection, and further processed by another residual block. In this way, the decoder combines the global contextual information learned by the Swin Transformer encoder with the local structural details preserved in shallow features. This combination is particularly beneficial for medical image segmentation, where both semantic consistency and boundary precision are important.

Finally, the segmentation prediction is generated by a 1×1×1 convolutional layer followed by a sigmoid activation function. Therefore, the entire network establishes an end-to-end mapping from the input volume X to the output segmentation $\hat{Y}=f\left(X\right)$, while the teacher and student branches differ only in their training roles and parameter update strategies rather than in network architecture.

### 3.2. Interpolation Consistency-Based Data Consistency Training

To alleviate the lack of supervision signals caused by sparse annotations, we introduce an interpolation consistency-based data consistency training strategy on unlabeled data [52,53]. The key idea is to construct mixed inputs and mixed soft pseudo-labels in the data space, so that the network is encouraged to produce consistent predictions under interpolation. In contrast to hard pseudo-labels, which may introduce overconfident and potentially incorrect supervision, soft pseudo-labels preserve uncertainty information and provide smoother optimization targets.

Let $u_{j},u_{k}\in D_{u}$ denote two unlabeled 3D MRI volumes sampled from the unlabeled set. Their corresponding teacher predictions are denoted by

(7) $$p_{j}=f_{t}\left(u_{j};\bar{\theta }\right),\;p_{k}=f_{t}\left(u_{k};\bar{\theta }\right),$$

where $p_{j}$ and $p_{k}$ are voxel-wise probability maps. We then define the interpolation operator ${\mathrm{Mix}}_{\lambda }\left(\cdot ,\cdot \right)$ as

(8) $${\mathrm{Mix}}_{\lambda }\left(a,b\right)=\lambda a+\left(1-\lambda \right)b,$$

where λ∈[0,1] is the mixture coefficient, randomly sampled during each training iteration.

Based on this operator, the mixed unlabeled input and the corresponding mixed soft pseudo-label are constructed as

(9) $${\tilde{u}}_{jk}={\mathrm{Mix}}_{\lambda }\left(u_{j},u_{k}\right),$$

(10) $${\tilde{p}}_{jk}={\mathrm{Mix}}_{\lambda }\left(p_{j},p_{k}\right)={\mathrm{Mix}}_{\lambda }\left(f_{t}\left(u_{j};\bar{\theta }\right),f_{t}\left(u_{k};\bar{\theta }\right)\right).$$

The student branch is then required to produce a prediction on the mixed input that is consistent with the mixed soft pseudo-label:

(11) $$f_{s}\left({\tilde{u}}_{jk};\theta \right)\approx {\tilde{p}}_{jk}.$$

This interpolation consistency can be equivalently written as

(12) $$f_{s}\left({\mathrm{Mix}}_{\lambda }\left(u_{j},u_{k}\right);\theta \right)\approx {\mathrm{Mix}}_{\lambda }\left(f_{t}\left(u_{j};\bar{\theta }\right),f_{t}\left(u_{k};\bar{\theta }\right)\right),$$

which encourages the network prediction at the interpolated sample to remain consistent with the interpolation of predictions at the original samples.

Let $B_{u}\subset D_{u}$ denote an unlabeled mini-batch, and let $B_{u}^{\mathrm{mix}}$ denote the set of sampled unlabeled pairs used for interpolation within this mini-batch. Accordingly, the semi-supervised data consistency loss is defined as

(13) $$L_{\mathrm{dc}}=\frac{1}{|B_{u}^{\mathrm{mix}}|}\sum_{\left(u_{j},u_{k}\right)\in B_{u}^{\mathrm{mix}}}\ell_{\mathrm{seg}}\left(f_{s}\left({\mathrm{Mix}}_{\lambda }\left(u_{j},u_{k}\right);\theta \right),\mathrm{stopgrad}\left[{\mathrm{Mix}}_{\lambda }\left(f_{t}\left(u_{j};\bar{\theta }\right),f_{t}\left(u_{k};\bar{\theta }\right)\right)\right]\right),$$

where $\ell_{\mathrm{seg}}\left(\cdot ,\cdot \right)$ denotes the segmentation loss function, and stopgrad(·) indicates that the mixed soft pseudo-label is detached from back-propagation.

This design increases the diversity of pseudo-supervision and reduces the tendency of the model to overfit to a single prediction bias. Moreover, we do not convert the mixed soft pseudo-label into a hard label using an argmax operation, since such a step would discard uncertainty information and may amplify the effect of incorrect pseudo-labels. By retaining the soft probabilistic target, the proposed strategy provides more informative supervision and improves the robustness of semi-supervised training on sparsely annotated medical images.

### 3.3. Mean Teacher-Based Model Consistency Training

In addition to interpolation consistency in the data space, we further introduce a mean teacher-based model consistency training strategy to provide stable supervision for unlabeled samples [54,55]. The purpose of this component is to enforce prediction consistency between the student and teacher branches for the same underlying unlabeled input, thereby improving the robustness and generalization of the semi-supervised segmentation framework. Compared with directly using the current student prediction as a pseudo-target, the teacher branch provides a temporally smoothed reference, which is less sensitive to short-term fluctuations during optimization.

For $u_{j}\in D_{u}$, the student branch receives a strongly augmented input in the teacher–student framework, while the teacher branch receives a weakly augmented input. Let $A_{s}\left(\cdot \right)$ and $A_{w}\left(\cdot \right)$ denote the strong and weak augmentation operators, respectively. The corresponding student and teacher predictions are written as

(14) $$p_{j}^{s}=f_{s}\left(A_{s}\left(u_{j}\right);\theta \right),\;$$

(15) $$p_{j}^{t}=f_{t}\left(A_{w}\left(u_{j}\right);\bar{\theta }\right),$$

where $p_{j}^{s}$ and $p_{j}^{t}$ are voxel-wise probability maps predicted by the student and teacher branches, respectively.

The teacher parameters are not optimized directly by back-propagation. Instead, they are updated as an exponential moving average (EMA) of the student parameters:

(16) $${\bar{\theta }}_{t}=\alpha {\bar{\theta }}_{t-1}+\left(1-\alpha \right)\theta_{t},$$

where *t* denotes the training iteration and α∈[0,1) is the EMA decay coefficient. This update mechanism enables the teacher branch to evolve more smoothly than the student branch and therefore provide more reliable pseudo-supervision during training.

Based on this formulation, model consistency requires the student prediction under strong augmentation to remain close to the teacher prediction under weak augmentation for the same unlabeled sample, i.e.,

(17) $$f_{s}\left(A_{s}\left(u_{j}\right);\theta \right)\approx f_{t}\left(A_{w}\left(u_{j}\right);\bar{\theta }\right).$$

Accordingly, the model consistency loss is defined as

(18) $$L_{\mathrm{mc}}=\frac{1}{|B_{u}|}\sum_{u_{j}\in B_{u}}\ell_{\mathrm{seg}}\left(f_{s}\left(A_{s}\left(u_{j}\right);\theta \right),\mathrm{stopgrad}\left[f_{t}\left(A_{w}\left(u_{j}\right);\bar{\theta }\right)\right]\right),\;$$

where $B_{u}$ is the unlabeled mini-batch defined above and stopgrad(·) indicates that gradients are not propagated through the teacher branch. In this way, the teacher model serves as a stable target generator, while the student model is optimized to match the teacher prediction under different augmentation strengths.

This mean teacher-based design improves the stability of pseudo-supervision by reducing the influence of noisy updates from individual iterations. Since the teacher parameters are obtained by temporal averaging rather than direct optimization, the resulting teacher predictions are generally smoother and more reliable than instantaneous student outputs. Consequently, model consistency complements the proposed data consistency strategy: the latter enforces interpolation consistency across different unlabeled samples, whereas the former enforces branch-level consistency for the same unlabeled sample under the teacher–student learning scheme.

### 3.4. Auxiliary Structure-Weighted Consistency Training

To further account for the irregular and heterogeneous morphology of brain tumors, we introduce an auxiliary structure-weighted consistency strategy. The key idea is to use a local complexity descriptor only as an auxiliary parameter that redistributes consistency supervision in unlabeled volumes, so that structurally complex regions receive stronger regularization. This design does not change the SwinUNeTR backbone or the teacher–student training scheme, but modifies the voxel-wise weighting of the consistency losses.

For an unlabeled volume *u*, let $\Phi \left(u\right)\in {\left[0,1\right]}^{H\times W\times D}$ denote its normalized local complexity map. In this work, Φ(u) is estimated from local multi-scale box-counting statistics on the edge response of *u* and is used only as an auxiliary weighting parameter. Based on Φ(u), the auxiliary weight map is defined as

(19) $$W\left(u\right)=N_{\mathrm{mean}}\left(w_{\min}1+\left(w_{\max}-w_{\min}\right)\Phi \left(u\right)\right),$$

where $W_{v}\left(u\right)$ denotes the voxel-wise auxiliary weight at voxel *v*, 1 is an all-one map with the same spatial size as Φ(u), $w_{\min}$ and $w_{\max}$ denote the lower and upper bounds of the auxiliary weight, and $N_{\mathrm{mean}}\left(\cdot \right)$ normalizes the weight map to have unit mean. This normalization keeps the global scale of the consistency objective stable while assigning relatively larger weights to locally complex regions.

For prediction *q*, target *r*, and weight map *W*, we define the weighted mean squared error as

(20) $$\ell_{\mathrm{wmse}}\left(q,r;W\right)=\frac{\sum_{v}W_{v}\frac{1}{K}\sum_{c=1}^{K}{\left(q_{v,c}-r_{v,c}\right)}^{2}}{\sum_{v}W_{v}},$$

where *K* denotes the number of prediction channels; for the binary segmentation setting in this work, K=1.

For two unlabeled samples $u_{j}$ and $u_{k}$, the mixed auxiliary weight map is constructed using the same interpolation operator as the mixed image and mixed soft pseudo-label:

(21) $${\tilde{W}}_{jk}={\mathrm{Mix}}_{\lambda }\left(W\left(u_{j}\right),W\left(u_{k}\right)\right).$$

Accordingly, the auxiliary weighted data consistency loss is defined as

(22) $$L_{\mathrm{dc}}^{\mathrm{aw}}=\frac{1}{|B_{u}^{\mathrm{mix}}|}\sum_{\left(u_{j},u_{k}\right)\in B_{u}^{\mathrm{mix}}}\ell_{\mathrm{wmse}}\left(f_{s}\left({\mathrm{Mix}}_{\lambda }\left(u_{j},u_{k}\right);\theta \right),\mathrm{stopgrad}\left[{\mathrm{Mix}}_{\lambda }\left(p_{j},p_{k}\right)\right];{\tilde{W}}_{jk}\right),$$

where $p_{j}=f_{t}\left(u_{j};\bar{\theta }\right)$ and $p_{k}=f_{t}\left(u_{k};\bar{\theta }\right)$ are the teacher-generated soft pseudo-labels defined in the interpolation consistency formulation.

The same auxiliary weighting principle is also applied to the model consistency term. For an unlabeled sample $u_{j}$, the auxiliary weighted model consistency loss is defined as

(23) $$L_{\mathrm{mc}}^{\mathrm{aw}}=\frac{1}{|B_{u}|}\sum_{u_{j}\in B_{u}}\ell_{\mathrm{wmse}}\left(p_{j}^{s},\mathrm{stopgrad}\left[p_{j}^{t}\right];W\left(u_{j}\right)\right),$$

where $p_{j}^{s}=f_{s}\left(A_{s}\left(u_{j}\right);\theta \right)$ and $p_{j}^{t}=f_{t}\left(A_{w}\left(u_{j}\right);\bar{\theta }\right)$ are the student and teacher predictions defined in the mean teacher formulation.

In this way, the proposed auxiliary weighting strategy preserves the original interpolation consistency and mean teacher mechanisms, while making both consistency terms sensitive to local structural complexity. Compared with uniform consistency regularization, the proposed strategy encourages stronger supervision around irregular tumor boundaries and heterogeneous lesion regions, which are the areas where accurate semi-supervised segmentation is often more difficult.

### 3.5. Objective

Based on the above formulations, the overall training objective consists of a supervised segmentation loss on labeled data and an auxiliary weighted semi-supervised loss on unlabeled data. The supervised term is used to learn reliable voxel-wise segmentation from annotated samples, while the semi-supervised term exploits unlabeled samples through weighted interpolation consistency and weighted model consistency.

For a labeled mini-batch $B_{l}\subset D_{l}$, the supervised loss is defined as

(24) $$L_{\sup}=\frac{1}{|B_{l}|}\sum_{\left(x_{i},y_{i}\right)\in B_{l}}\ell_{\mathrm{seg}}\left(f_{s}\left(x_{i};\theta \right),y_{i}\right),$$

where $\ell_{\mathrm{seg}}\left(\cdot ,\cdot \right)$ is the segmentation loss function defined above, here computed between the student prediction and the ground-truth annotation.

For unlabeled data, the semi-supervised objective is composed of the auxiliary weighted data consistency loss $L_{\mathrm{dc}}^{\mathrm{aw}}$ and the auxiliary weighted model consistency loss $L_{\mathrm{mc}}^{\mathrm{aw}}$. The overall auxiliary weighted semi-supervised loss is written as

(25) $$L_{\mathrm{semi}}^{\mathrm{aw}}=L_{\mathrm{dc}}^{\mathrm{aw}}+\beta L_{\mathrm{mc}}^{\mathrm{aw}},$$

where β is a weighting coefficient that controls the relative contribution of the weighted model consistency term.

Accordingly, the final training objective of the proposed framework is given by

(26) $$L=L_{\sup}+\lambda_{u}\left(t\right)L_{\mathrm{semi}}^{\mathrm{aw}}=L_{\sup}+\lambda_{u}\left(t\right)\left(L_{\mathrm{dc}}^{\mathrm{aw}}+\beta L_{\mathrm{mc}}^{\mathrm{aw}}\right),$$

where $\lambda_{u}\left(t\right)$ is a time-dependent trade-off coefficient for the semi-supervised objective. To improve training stability and reproducibility, $\lambda_{u}\left(t\right)$ is explicitly initialized as zero at the beginning of training:

(27) $$\lambda_{u}\left(0\right)=0.$$

This initialization prevents unreliable early teacher predictions and noisy pseudo-supervision from dominating the optimization before the student network has learned a reliable supervised representation from the labeled data.

In our implementation, $\lambda_{u}\left(t\right)$ follows a linear warm-up schedule:

(28) $$\lambda_{u}\left(t\right)=\left\{\begin{array}{cc} \lambda_{\max}\frac{t}{T_{r}}, & 0\leq t<T_{r},\\ \lambda_{\max}, & t\geq T_{r}, \end{array}\right.$$

where $T_{r}$ denotes the ramp-up length and $\lambda_{\max}$ denotes the maximum weight of the semi-supervised objective. Unless otherwise specified, we set $\lambda_{\max}=1.0$ and $T_{r}=5000$ iterations. Therefore, the training process starts from a supervised-dominant stage and gradually introduces the auxiliary weighted consistency terms as the teacher predictions become more reliable.

The increase of $\lambda_{u}\left(t\right)$ is not based on a manually adjusted threshold. Instead, it is controlled by the deterministic warm-up schedule above. During this warm-up stage, training stability is monitored using the moving averages of the supervised loss $L_{\sup}$, the semi-supervised consistency loss $L_{\mathrm{semi}}^{\mathrm{aw}}$, and the teacher–student model consistency loss $L_{\mathrm{mc}}^{\mathrm{aw}}$, together with the validation Dice score. These quantities are used to verify that the supervised learning signal decreases smoothly and that the teacher–student predictions do not show divergent behaviour before the semi-supervised term reaches its full weight.

Since the auxiliary weight maps are normalized to have unit mean, the proposed objective does not simply increase the global scale of the consistency loss. Instead, it redistributes consistency supervision toward locally complex and boundary-irregular regions, encouraging the model to learn more stable predictions for heterogeneous tumor structures under limited annotation.

## 4. Experiments

### 4.1. Implementation Details

All experiments were implemented in PyTorch 2.7.1 and conducted on a workstation equipped with an NVIDIA GeForce RTX 3090 GPU (NVIDIA Corporation, Santa Clara, CA, USA) and an Intel Core i9-10900K CPU (Intel Corporation, Santa Clara, CA, USA). For a fair comparison, all baseline methods and the proposed method were trained under the same experimental protocol. Specifically, all 3D volumes were resized to 96×96×96, the batch size was set to 2, and each model was trained for 30,000 iterations. Unless otherwise specified, the model parameters were optimized using SGD with an initial learning rate of 0.01, a momentum of 0.9, and a weight decay of $1\times 10^{-4}$. For the teacher–student training scheme, the EMA decay coefficient was set to α=0.99, and the relative weight of the model consistency term was set to β=1.0. The semi-supervised trade-off coefficient $\lambda_{u}\left(t\right)$ was initialized as 0 and linearly ramped up to $\lambda_{\max}=1.0$ during the first $T_{r}=5000$ iterations. After the ramp-up stage, $\lambda_{u}\left(t\right)$ remained fixed at 1.0 for the rest of training. During training, we monitored the 100-iteration moving averages of $L_{\sup}$, $L_{\mathrm{semi}}^{\mathrm{aw}}$, and $L_{\mathrm{mc}}^{\mathrm{aw}}$, as well as the validation Dice score every 1000 iterations, to ensure that the semi-supervised term was introduced after the supervised optimization became stable. The model achieving the best performance on the validation set was selected for final testing. For the auxiliary weighting settings, the local complexity map was computed using box-counting scales S={3,5,9}. The edge threshold parameter was set to τ=0.5, and the auxiliary weight bounds were set to $w_{\min}=0.5$ and $w_{\max}=2.0$.

### 4.2. Dataset

The proposed semi-supervised learning method was evaluated on the MICCAI Brain Tumor Segmentation (BraTS) 2019 dataset [56]. The dataset contains routine clinically acquired multimodal MRI scans with voxel-wise ground-truth annotations provided by expert neuroradiologists. In each experiment, 80% of the cases were used for training and the remaining 20% were used for testing.

To evaluate the proposed method under different annotation conditions, we further divided the training set into labeled and unlabeled subsets with different ratios. Specifically, four labeled-data proportions were considered: 10%, 20%, 40%, and 80% of the training cases. The remaining training cases were treated as unlabeled data and were used only through the semi-supervised learning objective. Therefore, the corresponding labeled/unlabeled settings were 10%/90%, 20%/80%, 40%/60%, and 80%/20%, respectively. This design enables a systematic evaluation of the proposed framework across different annotation budgets, ranging from highly annotation-scarce settings to relatively label-rich settings.

### 4.3. Baseline Methods

To provide a systematic evaluation, the baseline methods are organized from two complementary perspectives: network backbone and semi-supervised training strategy.

For the network backbone comparison, we consider three representative 3D segmentation architectures. The first is 3D U-Net, which represents the conventional CNN-based encoder–decoder framework for volumetric medical image segmentation [1,18]. The second is UNETR, which represents a Vision Transformer-based segmentation architecture that uses Transformer encoders for volumetric representation learning [57]. The third is SwinUNeTR, which introduces shifted-window self-attention into a hierarchical U-shaped framework and serves as the backbone adopted by the proposed method [51]. This backbone comparison allows us to evaluate the effect of moving from convolutional architectures to Transformer-based and Swin Transformer-based volumetric segmentation networks.

For the semi-supervised learning strategy comparison, we compare the proposed dual-consistency training framework with several representative SSL methods, including DAN [27], ADVENT [58], UAMT [59], and CPS [60]. These methods cover different semi-supervised learning principles, including adversarial learning, entropy-based regularization, uncertainty-aware teacher–student learning, and cross pseudo-supervision. To ensure a fair and comprehensive comparison, each SSL strategy is evaluated with the above three backbone families whenever applicable, namely 3D U-Net, UNETR, and SwinUNeTR. In this way, the experiments assess not only whether the proposed Semi-SwinUNeTR outperforms existing SSL strategies, but also whether the proposed dual-consistency design provides consistent benefits when combined with the SwinUNeTR backbone.

### 4.4. Metrics

Since brain tumor boundaries are often irregular and may exhibit complex geometry, boundary-sensitive metrics are important for evaluating whether a segmentation method can preserve complex lesion contours. Therefore, in addition to region-based metrics, we also report ASD and HD_95_ to assess surface-level segmentation accuracy.

To comprehensively evaluate segmentation performance, we adopt both region-based and boundary-based metrics. The region-based metrics include Dice, Accuracy, Precision, Sensitivity, Specificity, and Relative Absolute Volume Difference (RAVD), while the boundary-based metrics include Average Surface Distance (ASD) and the 95th percentile Hausdorff Distance (HD_95_). The region-based metrics are defined as follows:

(29) $$\mathrm{Dice}=\frac{2\times \mathrm{TP}}{2\times \mathrm{TP}+\mathrm{FP}+\mathrm{FN}},\;$$

(30) $$\mathrm{Accuracy}=\frac{\mathrm{TP}+\mathrm{TN}}{\mathrm{TP}+\mathrm{TN}+\mathrm{FP}+\mathrm{FN}},$$

(31) $$\mathrm{Precision}=\frac{\mathrm{TP}}{\mathrm{TP}+\mathrm{FP}},$$

(32) $$\mathrm{Sensitivity}=\frac{\mathrm{TP}}{\mathrm{TP}+\mathrm{FN}},$$

(33) $$\mathrm{Specificity}=\frac{\mathrm{TN}}{\mathrm{TN}+\mathrm{FP}},$$

(34) $$\mathrm{RAVD}=\frac{\left|\mathrm{FP}-\mathrm{FN}\right|}{\mathrm{TP}+\mathrm{FN}}.\;$$

In the above formulas, TP, TN, FP, and FN denote the numbers of true positives, true negatives, false positives, and false negatives, respectively.

For the boundary-based metrics, the distance from a voxel *x* to a voxel set *A* is defined as

(35) $$d\left(x,A\right)=\min_{y\in A}\mathrm{dist}\left(x,y\right),$$

where dist(x,y) denotes the Euclidean distance between two voxels while taking the actual image spacing into account. Based on this definition, ASD is computed as

(36) $$\mathrm{ASD}=\frac{\sum_{x\in \partial G}d\left(x,\partial M\right)+\sum_{y\in \partial M}d\left(y,\partial G\right)}{\left|\partial G|+|\partial M\right|},$$

where *M* and *G* denote the predicted segmentation and the ground truth, respectively, and ∂M and ∂G denote their corresponding surfaces. The 95th percentile Hausdorff Distance is computed from the set of bidirectional surface distances:

(37) $$D_{\mathrm{surf}}=\left\{d(x,\partial M)|x\in \partial G\right\}\cup \left\{d(y,\partial G)|y\in \partial M\right\},\;$$

(38) $${\mathrm{HD}}_{95}={\mathrm{Percentile}}_{95}\left(D_{\mathrm{surf}}\right).$$

### 4.5. Qualitative Results

**Figure 2:**
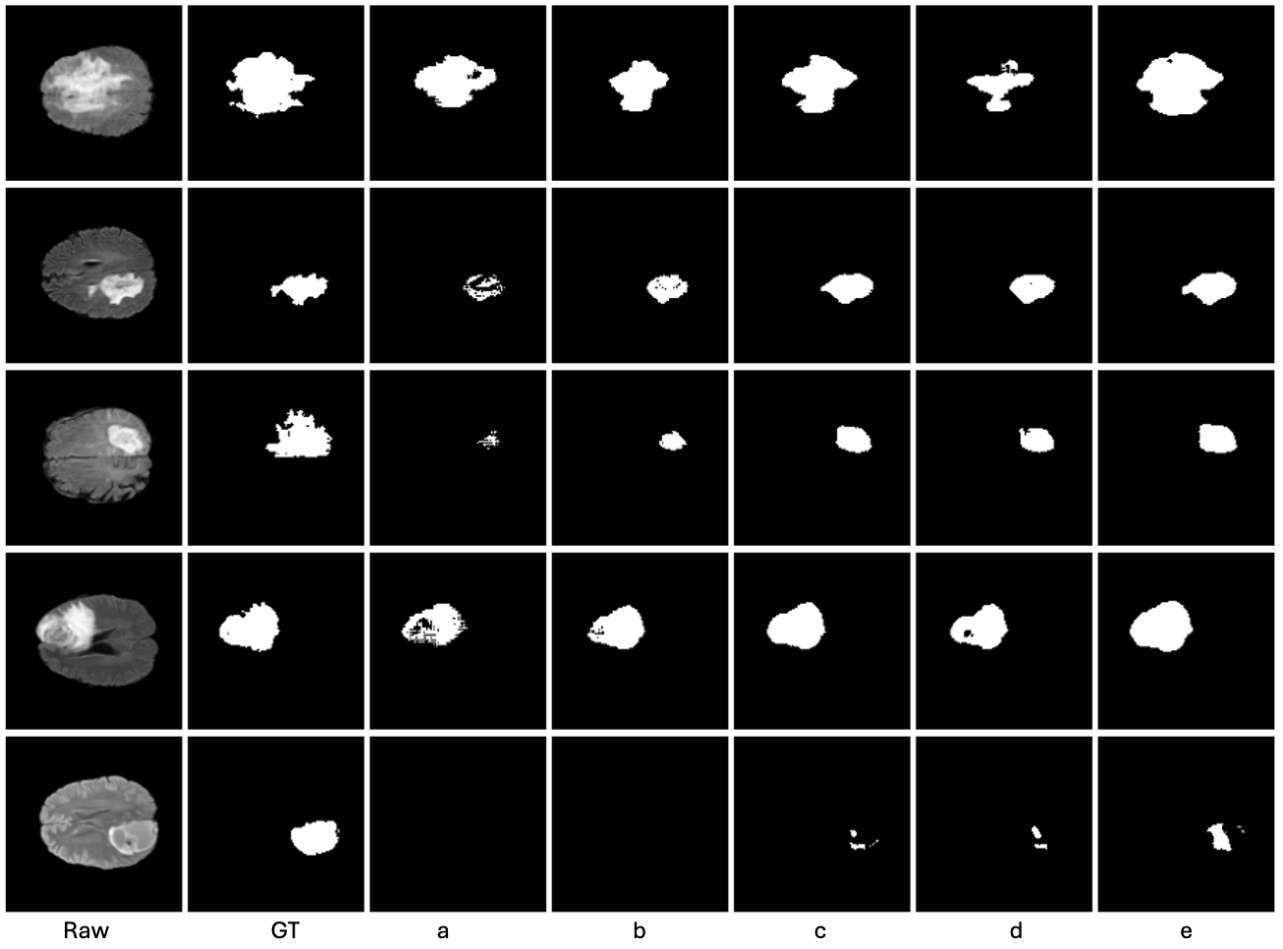
The Qualitative results on Brats dataset when 10% of training set is annotated, including Raw Images, Ground Truth, (**a**) DAN, (**b**) ADVENT, (**c**) UAMt, (**d**) CPS, and (**e**) ours.

**Figure 3:**
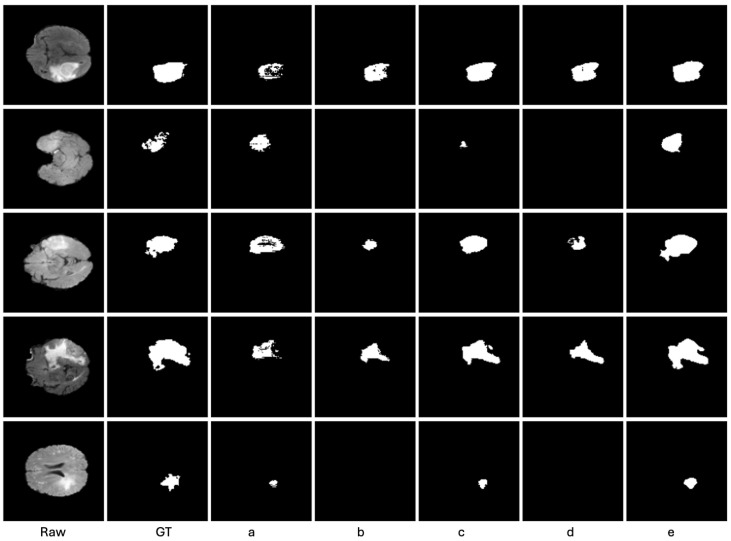
The Qualitative results on Brats dataset when 20% of training set is annotated, including Raw Images, Ground Truth, (**a**) DAN, (**b**) ADVENT, (**c**) UAMt, (**d**) CPS, and (**e**) ours.

Representative qualitative results are presented in Figure 2 and Figure 3. Figure 2 shows the segmentation results when only 10% of the training set is annotated, while Figure 3 reports the corresponding results under the 20% labeled-data setting. In each figure, the first two columns show the raw MRI images and the ground-truth masks, followed by the predictions generated by different semi-supervised methods, including DAN, ADVENT, UAMT, CPS, and the proposed method.

As shown in Figure 2 and Figure 3, the proposed Semi-SwinUNeTR produces segmentation masks that are visually closer to the ground truth. Compared with the competing methods, our method better preserves the overall tumor structure and generates more complete lesion regions. In several challenging cases, the baseline methods either miss part of the tumor region or produce fragmented predictions, whereas the proposed method yields more coherent and spatially consistent segmentation results. These qualitative observations are consistent with the quantitative results and further demonstrate the effectiveness of combining SwinUNeTR with model consistency and data consistency training under limited annotation.

### 4.6. Quantitative Results

**Figure 4:**
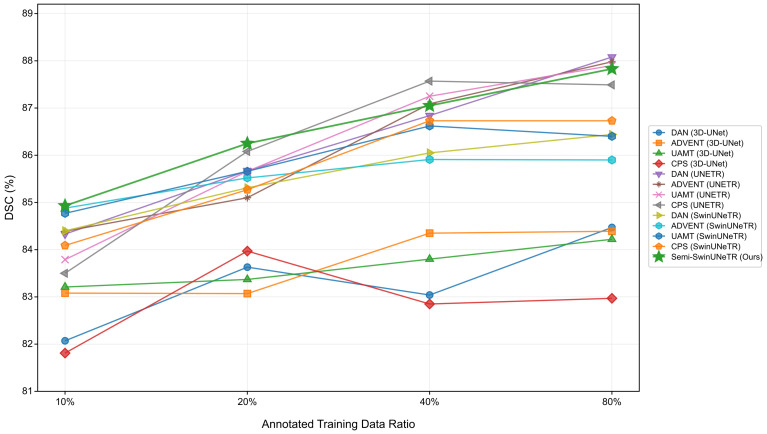
Comparison of Dice under different annotation ratios. Dice scores are compared across different semi-supervised learning methods under increasing annotation ratios on the brain tumor MRI testing set. The proposed Semi-SwinUNeTR is highlighted in green and shows strong performance under limited annotation settings, particularly when only 10% and 20% of the training data are annotated.

As shown in Figure 4, the Dice scores generally increase as the proportion of annotated training data rises from 10% to 80%, indicating that all methods benefit from additional labelled data. The proposed Semi-SwinUNeTR achieves the highest Dice score under the 10% and 20% annotation settings, with DSC values of 84.93% and 86.25%, respectively. This suggests that the proposed method is particularly effective in low-annotation scenarios. When the annotation ratio increases to 40% and 80%, Semi-SwinUNeTR remains highly competitive, achieving 87.05% and 87.83%, respectively, and stays close to the best-performing baselines. Overall, the results demonstrate that Semi-SwinUNeTR provides stable and strong segmentation performance across different levels of annotation availability.

Table 1, Table 2, Table 3 and Table 4 report the quantitative comparison under four annotation settings, where 10%, 20%, 40%, and 80% of the training cases are labeled, respectively. The comparison is organized across both different semi-supervised learning strategies and different backbone architectures, including 3D U-Net, UNETR, and SwinUNeTR. Overall, the results show that the proposed Semi-SwinUNeTR is particularly effective in low-label regimes and remains consistently strong across different annotation budgets.

When only 10% of the training set is annotated, as shown in Table 1, the proposed Semi-SwinUNeTR achieves a Dice score of 0.8493, outperforming the compared semi-supervised baselines. It also obtains strong boundary-related performance, with RAVD, HD_95_, and ASD values of 0.3334, 9.0079, and 2.0790, respectively. Compared with the SwinUNeTR baselines trained with existing SSL strategies, the proposed method further improves both region-based and boundary-based performance, demonstrating the benefit of combining SwinUNeTR with the proposed dual-consistency training strategy. After incorporating the auxiliary weighting parameter, Semi-SwinUNeTR (Weighted) further improves the Dice score from 0.8493 to 0.8564 and increases Accuracy, Precision, and Specificity to 0.9934, 0.9109, and 0.9980, respectively. It also reduces RAVD, HD_95_, and ASD to 0.3243, 8.9826, and 2.0475, indicating better volume agreement and boundary accuracy. These results suggest that the auxiliary weighting parameter can provide additional structural guidance for low-label segmentation by emphasizing irregular and boundary-complex tumor regions. When the labeled-data ratio increases to 20%, as reported in Table 2, Semi-SwinUNeTR continues to provide strong overall performance. It achieves a Dice score of 0.8625, with Accuracy and Sensitivity values of 0.9911 and 0.8249, respectively. It also obtains strong boundary-related performance, with RAVD, HD_95_, and ASD values of 0.2994, 8.2974, and 2.0637, respectively. After incorporating the auxiliary weighting parameter, Semi-SwinUNeTR (Weighted) further improves the Dice score to 0.8794 and increases Accuracy, Precision, Sensitivity, and Specificity to 0.9927, 0.9085, 0.8521, and 0.9972, respectively. It also reduces RAVD and HD_95_ to 0.2953 and 8.1854, indicating improved volume agreement and reduced boundary outliers. Although its ASD value of 2.0864 is slightly higher than that of Semi-SwinUNeTR, the overall results show that this weighting term provides additional benefits when more labeled data become available.

For the 40% labeled-data setting in Table 3, the performance gap between different methods becomes smaller. Several UNETR-based methods obtain slightly higher Dice scores, while the proposed Semi-SwinUNeTR remains the best-performing method among the SwinUNeTR-based variants. In particular, Semi-SwinUNeTR achieves a Dice score of 0.8705 and an HD_95_ value of 8.3575, indicating strong boundary accuracy. Semi-SwinUNeTR (Weighted) obtains a Dice score of 0.8651, which is slightly lower than the original Semi-SwinUNeTR, but it improves Accuracy, Precision, and Specificity to 0.9928, 0.9164, and 0.9978, respectively. It also reduces RAVD from 0.2956 to 0.2843 and ASD from 2.0555 to 2.0477, while maintaining a comparable HD_95_ value of 8.3621. These results suggest that, under a moderate annotation budget, the auxiliary weighting term mainly improves prediction precision, volume agreement, and average surface accuracy, although the gain in Dice is less consistent.

When 80% of the training set is annotated, as shown in Table 4, the differences among strong transformer-based methods become relatively small. Some UNETR-based baselines achieve competitive boundary-related results, but the proposed Semi-SwinUNeTR remains the strongest method within the SwinUNeTR family. It achieves Dice, Accuracy, Sensitivity, RAVD, HD_95_, and ASD values of 0.8783, 0.9920, 0.8570, 0.2846, 7.5725, and 1.9379, respectively. With the auxiliary weighting parameter, Semi-SwinUNeTR (Weighted) further improves Dice, Accuracy, Precision, and Specificity to 0.8859, 0.9946, 0.9176, and 0.9981, respectively, while slightly decreasing Sensitivity to 0.8563. It also reduces RAVD, HD_95_, and ASD to 0.2743, 7.4533, and 1.8950, respectively. These results indicate that the auxiliary weighting term remains useful in relatively label-rich settings by improving region overlap, volume-level agreement, and boundary accuracy without changing the SwinUNeTR backbone.

The stable performance of Semi-SwinUNeTR across annotation ratios also suggests that the proposed framework can effectively learn complex multi-scale tumor morphology from partially labeled data. This is important for biomedical images where lesion boundaries are irregular and local structural variations may not be sufficiently captured by purely local or label-intensive training strategies. Taken together, the quantitative results demonstrate three main observations. First, Semi-SwinUNeTR shows clear advantages when labeled data are scarce, especially under the 10% and 20% annotation settings. Second, the proposed method consistently improves the SwinUNeTR backbone across all annotation ratios, confirming the effectiveness of the proposed model consistency and data consistency mechanisms. Third, as the amount of labeled data increases, the overall performance gap among strong transformer-based methods becomes narrower, but Semi-SwinUNeTR still maintains stable and competitive segmentation accuracy. These findings support the effectiveness of combining a SwinUNeTR backbone with dual-consistency semi-supervised learning for annotation-efficient 3D brain tumor segmentation.

To further assess whether the observed improvements are statistically meaningful, we conducted paired Wilcoxon signed-rank tests using case-wise Dice scores on the test set. The proposed method was compared with the strongest corresponding baseline under each annotation ratio. The results show that the improvements are statistically significant, with all *p*-values smaller than 0.0005. This confirms that the proposed consistency learning strategy provides reliable and significant performance gains across different annotation settings.

### 4.7. Ablation Study

**Figure 5:**
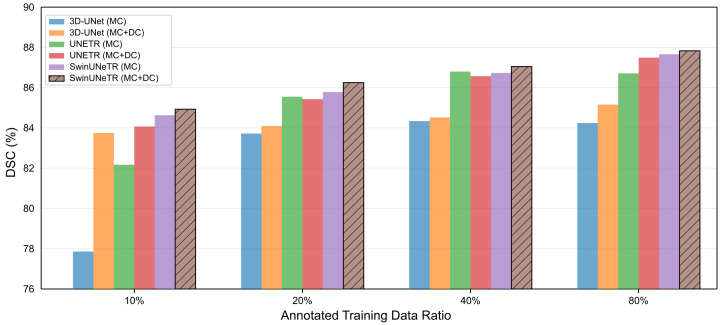
Dice comparison when the annotated training data are gradually increased. The figure compares different backbone and consistency configurations under different annotation ratios. MC denotes model consistency, DC denotes data consistency, and the hatched bars indicate the proposed configuration, SwinUNeTR with both MC and DC.

Figure 5 provides an overall visual comparison of the ablation results, while Table 5, Table 6, Table 7 and Table 8 report the detailed numerical results under different annotation ratios.

In Figure 5, MC denotes model consistency and DC denotes data consistency. For each annotation ratio, we compare three backbone architectures, namely 3D U-Net, UNETR, and SwinUNeTR, under the model-consistency-only setting and the combined model-consistency and data-consistency setting. The hatched bars correspond to the proposed Semi-SwinUNeTR configuration, where SwinUNeTR is trained with both MC and DC.

Table 5, Table 6, Table 7, Table 8 and Table 9 report the ablation results under different annotation ratios.

When only 10% of the training set is labeled, as shown in Table 5, adding data consistency improves the Dice score for all three backbones. For 3D U-Net, the Dice score increases from 0.7786 to 0.8375. For UNETR, it increases from 0.8217 to 0.8407. For SwinUNeTR, it further improves from 0.8463 to 0.8493. These results indicate that the proposed data consistency term provides useful additional supervision when labeled data are highly limited. The improvement is particularly large for 3D U-Net, while SwinUNeTR already provides a strong model-consistency baseline and still benefits from the additional data consistency term.

At the 20% labeled-data setting in Table 6, data consistency continues to improve 3D U-Net and SwinUNeTR. The Dice score of 3D U-Net increases from 0.8372 to 0.8410, and that of SwinUNeTR increases from 0.8578 to 0.8625. For UNETR, the Dice score changes slightly from 0.8555 to 0.8543. Although the gain is not uniform across all backbones, SwinUNeTR equipped with both model consistency and data consistency achieves the best Dice score among all ablation variants at this annotation ratio.

At the 40% labeled-data setting in Table 7, the effect of data consistency remains positive for 3D U-Net and SwinUNeTR. The Dice score of 3D U-Net increases from 0.8434 to 0.8452, while the Dice score of SwinUNeTR increases from 0.8673 to 0.8705. For UNETR, the Dice score slightly decreases from 0.8680 to 0.8657. Nevertheless, SwinUNeTR with both consistency mechanisms achieves the highest Dice score in this setting, suggesting that the proposed data consistency strategy is particularly compatible with the SwinUNeTR backbone.

When 80% of the training set is labeled, as reported in Table 8, adding data consistency improves all three backbones. The Dice score increases from 0.8424 to 0.8516 for 3D U-Net, from 0.8671 to 0.8749 for UNETR, and from 0.8766 to 0.8783 for SwinUNeTR. Although the absolute gain for SwinUNeTR is smaller than that for the other backbones, SwinUNeTR with both model consistency and data consistency still achieves the best Dice score among all ablation variants.

Overall, the ablation results show that the proposed data consistency strategy provides complementary supervision on top of the mean teacher-based model consistency framework. Across all four annotation ratios, SwinUNeTR with both model consistency and data consistency consistently achieves the best Dice performance among the compared ablation variants. These results verify the effectiveness of combining the SwinUNeTR backbone with the proposed dual-consistency semi-supervised training strategy.

## 5. Conclusions

This paper introduced Semi-SwinUNeTR, a semi-supervised framework for 3D brain tumor segmentation with limited annotation. The method integrates a Swin Transformer-based U-shaped segmentation backbone with two complementary consistency learning strategies: interpolation-based data consistency and mean teacher-based model consistency. By jointly exploiting labeled and unlabeled data, the proposed framework improves annotation efficiency while preserving the long-range contextual modelling capability of SwinUNeTR. To further address the irregular and heterogeneous morphology of brain tumors, an auxiliary local complexity parameter is used as voxel-wise weighting in the consistency objective. This design encourages stronger supervision in structurally complex and boundary-irregular regions without changing the underlying SwinUNeTR backbone.

Experiments on the BraTS 2019 benchmark show that Semi-SwinUNeTR is particularly effective when annotated data are scarce. The proposed method achieves the best Dice performance under the 10% and 20% annotation settings and remains highly competitive as the annotation ratio increases. The comparative results further indicate that combining model consistency with data consistency brings consistent improvements over model consistency alone, confirming the complementary role of the proposed data-level regularization. These findings suggest that the semi-supervised strategy is well matched with the SwinUNeTR architecture and can improve segmentation robustness under different levels of annotation availability.

Overall, Semi-SwinUNeTR provides a practical and effective approach for reducing the dependence on dense manual annotation in 3D medical image segmentation. The results suggest that hierarchical Swin Transformer representations and dual-consistency regularization are useful for modelling irregular, heterogeneous, and multi-scale lesion structures under limited annotation. By incorporating the auxiliary weighting parameter, Semi-SwinUNeTR (Weighted) further improves the overall performance in most annotation settings, especially under the 10%, 20%, and 80% labeled-data ratios, where it achieves higher Dice scores and better boundary-related metrics than the original Semi-SwinUNeTR. The results indicate that local structural weighting can provide effective guidance by redistributing consistency supervision toward irregular and boundary-complex tumor regions without changing the SwinUNeTR backbone. Future work will investigate more robust local structure descriptors and cross-dataset validation for multi-class tumor subregion segmentation.

## Figures and Tables

**Table 1:** The Direct Comparison Between Each SSL Method on Brain Tumor MRI Testing Set When 10% of Training Set is Annotated. The upward and downward arrows indicate that larger and smaller values are better, respectively.

Framework	Network	Dice ↑	Acc ↑	Pre ↑	Sen ↑	Spe ↑	RAVD ↓	HD_95_ ↓	ASD ↓
DAN [27]	3D-UNet	0.8207	0.9885	0.8684	0.7779	0.9959	0.5675	11.8804	2.3767
ADVENT [58]	3D-UNet	0.8308	0.9894	0.8985	0.7726	0.9970	0.6319	10.5194	2.7100
UAMT [59]	3D-UNet	0.8321	0.9894	0.8880	0.7828	0.9966	0.8822	11.6580	2.8493
CPS [60]	3D-UNet	0.8181	0.9887	0.8967	0.7521	0.9970	0.5457	14.9031	2.5704
DAN [27]	UNETR	0.8433	0.9901	0.9061	0.7886	0.9972	0.3768	12.3983	2.2703
ADVENT [58]	UNETR	0.8439	0.9900	0.8912	0.8014	0.9966	0.3352	12.2276	2.2175
UAMT [59]	UNETR	0.8379	0.9897	0.8916	0.7903	0.9966	0.3648	17.8812	2.2411
CPS [60]	UNETR	0.8350	0.9897	0.9097	0.7717	0.9973	0.4126	11.3065	2.4244
DAN [27]	SwinUNeTR	0.8440	0.9901	0.8996	0.7949	0.9969	0.3906	9.0768	2.3504
ADVENT [58]	SwinUNeTR	0.8488	0.9903	0.8962	0.8062	0.9967	0.3587	9.0680	2.2429
UAMT [59]	SwinUNeTR	0.8477	0.9902	0.8928	0.8069	0.9966	0.3596	9.7649	2.2888
CPS [60]	SwinUNeTR	0.8409	0.9899	0.9009	0.7883	0.9970	0.4365	9.2517	2.4534
Semi-SwinUNeTR	SwinUNeTR	0.8493	0.9903	0.8927	0.8100	0.9966	0.3334	9.0079	2.0790
Semi-SwinUNeTR (Weighted)	SwinUNeTR	0.8564	0.9934	0.9109	0.8081	0.9980	0.3243	8.9826	2.0475

**Table 2:** The Direct Comparison Between Each SSL Method on Brain Tumor MRI Testing Set When 20% of Training Set is Annotated. The upward and downward arrows indicate that larger and smaller values are better, respectively.

Framework	Network	Dice ↑	Acc ↑	Pre ↑	Sen ↑	Spe ↑	RAVD ↓	HD_95_ ↓	ASD ↓
DAN [27]	3D-UNet	0.8363	0.9895	0.8800	0.7967	0.9962	0.4615	10.3074	2.3305
ADVENT [58]	3D-UNet	0.8307	0.9895	0.9099	0.7641	0.9974	0.5759	11.0624	2.5518
UAMT [59]	3D-UNet	0.8337	0.9895	0.8935	0.7815	0.9968	0.4834	9.2591	2.4863
CPS [60]	3D-UNet	0.8397	0.9899	0.8981	0.7885	0.9969	0.4651	9.8931	2.3543
DAN [27]	UNETR	0.8567	0.9908	0.9057	0.8126	0.9971	0.3283	8.4871	2.2372
ADVENT [58]	UNETR	0.8510	0.9906	0.9078	0.8008	0.9972	0.3639	9.4109	2.2559
UAMT [59]	UNETR	0.8566	0.9908	0.8975	0.8193	0.9967	0.3626	8.9823	2.0832
CPS [60]	UNETR	0.8608	0.9910	0.9004	0.8244	0.9968	0.3120	9.3036	2.1251
DAN [27]	SwinUNeTR	0.8531	0.9908	0.9173	0.7974	0.9975	0.3653	9.4717	2.2353
ADVENT [58]	SwinUNeTR	0.8552	0.9907	0.9014	0.8135	0.9969	0.3347	9.3015	2.2606
UAMT [59]	SwinUNeTR	0.8562	0.9907	0.8940	0.8215	0.9966	0.3449	8.7939	2.1745
CPS [60]	SwinUNeTR	0.8527	0.9906	0.9051	0.8060	0.9971	0.3859	8.6226	2.2063
Semi-SwinUNeTR	SwinUNeTR	0.8625	0.9911	0.9037	0.8249	0.9969	0.2994	8.2974	2.0637
Semi-SwinUNeTR (Weighted)	SwinUNeTR	0.8794	0.9927	0.9085	0.8521	0.9972	0.2953	8.1854	2.0864

**Table 3:** The Direct Comparison Between Each SSL Method on Brain Tumor MRI Testing Set When 40% of Training Set is Annotated. The upward and downward arrows indicate that larger and smaller values are better, respectively.

Framework	Network	Dice ↑	Acc ↑	Pre ↑	Sen ↑	Spe ↑	RAVD ↓	HD_95_ ↓	ASD ↓
DAN [27]	3D-UNet	0.8304	0.9894	0.9017	0.7695	0.9971	0.5308	9.9424	2.5637
ADVENT [58]	3D-UNet	0.8435	0.9900	0.8872	0.8039	0.9964	0.4559	9.4271	2.3345
UAMT [59]	3D-UNet	0.8380	0.9897	0.8946	0.7882	0.9968	0.5004	9.6191	2.4809
CPS [60]	3D-UNet	0.8285	0.9894	0.9146	0.7572	0.9975	0.6137	10.3741	2.6555
DAN [27]	UNETR	0.8684	0.9916	0.9137	0.8273	0.9973	0.2712	10.4967	2.1733
ADVENT [58]	UNETR	0.8709	0.9916	0.9073	0.8374	0.9970	0.2512	10.3208	2.0798
UAMT [59]	UNETR	0.8725	0.9918	0.9153	0.8335	0.9973	0.2693	8.4592	1.9202
CPS [60]	UNETR	0.8757	0.9920	0.9253	0.8311	0.9977	0.2454	8.9828	1.9695
DAN [27]	SwinUNeTR	0.8605	0.9911	0.9141	0.8129	0.9973	0.3215	8.5390	2.2793
ADVENT [58]	SwinUNeTR	0.8591	0.9909	0.9029	0.8195	0.9969	0.3590	8.4288	2.2271
UAMT [59]	SwinUNeTR	0.8661	0.9914	0.9109	0.8256	0.9972	0.3116	8.6247	2.1640
CPS [60]	SwinUNeTR	0.8678	0.9914	0.8984	0.8392	0.9967	0.3120	8.4861	2.1002
Semi-SwinUNeTR	SwinUNeTR	0.8705	0.9916	0.9042	0.8392	0.9969	0.2956	8.3575	2.0555
Semi-SwinUNeTR (Weighted)	SwinUNeTR	0.8651	0.9928	0.9164	0.8192	0.9978	0.2843	8.3621	2.0477

**Table 4:** The Direct Comparison Between Each SSL Method on Brain Tumor MRI Testing Set When 80% of Training Set is Annotated. The upward and downward arrows indicate that larger and smaller values are better, respectively.

Framework	Network	Dice ↑	Acc ↑	Pre ↑	Sen ↑	Spe ↑	RAVD ↓	HD_95_ ↓	ASD ↓
DAN [27]	3D-UNet	0.8447	0.9900	0.8908	0.8031	0.9966	0.3987	13.4456	2.0718
ADVENT [58]	3D-UNet	0.8439	0.9900	0.8940	0.7990	0.9967	0.4498	9.6865	2.3326
UAMT [59]	3D-UNet	0.8422	0.9900	0.9030	0.7891	0.9970	0.5543	8.7795	2.4636
CPS [60]	3D-UNet	0.8297	0.9895	0.9123	0.7608	0.9975	0.6167	11.9965	2.6493
DAN [27]	UNETR	0.8808	0.9922	0.9113	0.8523	0.9971	0.2410	7.0392	1.8754
ADVENT [58]	UNETR	0.8798	0.9922	0.9114	0.8504	0.9971	0.2495	10.0336	1.9568
UAMT [59]	UNETR	0.8790	0.9921	0.9081	0.8518	0.9970	0.2569	6.9889	1.9538
CPS [60]	UNETR	0.8749	0.9918	0.9003	0.8510	0.9967	0.2348	9.5645	1.8688
DAN [27]	SwinUNeTR	0.8644	0.9913	0.9146	0.8194	0.9973	0.3902	8.8914	2.5645
ADVENT [58]	SwinUNeTR	0.8590	0.9910	0.9148	0.8097	0.9974	0.7248	9.0524	2.6319
UAMT [59]	SwinUNeTR	0.8640	0.9914	0.9216	0.8131	0.9976	0.4989	8.4804	2.6642
CPS [60]	SwinUNeTR	0.8673	0.9913	0.8939	0.8422	0.9965	0.3599	7.6246	2.0601
Semi-SwinUNeTR	SwinUNeTR	0.8783	0.9920	0.9008	0.8570	0.9968	0.2846	7.5725	1.9379
Semi-SwinUNeTR (Weighted)	SwinUNeTR	0.8859	0.9946	0.9176	0.8563	0.9981	0.2743	7.4533	1.8950

**Table 5:** Ablation Studies on Contributions of Architecture and Modules on Brain Tumor MRI Testing Set When 10% of Training Set is Annotated. Checkmarks indicate that the corresponding component is included.

Model Consistency	Data Consistency	Network	Dice	Acc	Pre	Sen	Spe
✓		3D-UNet	0.7786	0.9859	0.8238	0.7382	0.9945
✓	✓	3D-UNet	0.8375	0.9897	0.8957	0.7865	0.9968
✓		UNETR	0.8217	0.9885	0.8581	0.7883	0.9955
✓	✓	UNETR	0.8407	0.9898	0.8884	0.7979	0.9965
✓		SwinUNeTR	0.8463	0.9902	0.8975	0.8006	0.9968
✓	✓	SwinUNeTR	0.8493	0.9903	0.8927	0.8100	0.9966

**Table 6:** Ablation Studies on Contributions of Architecture and Modules on Brain Tumor MRI Testing Set When 20% of Training Set is Annotated. Checkmarks indicate that the corresponding component is included.

Model Consistency	Data Consistency	Network	Dice	Acc	Pre	Sen	Spe
✓		3D-UNet	0.8372	0.9896	0.8823	0.7965	0.9963
✓	✓	3D-UNet	0.8410	0.9901	0.9111	0.7809	0.9973
✓		UNETR	0.8555	0.9908	0.9076	0.8090	0.9971
✓	✓	UNETR	0.8543	0.9907	0.9018	0.8116	0.9969
✓		SwinUNeTR	0.8578	0.9909	0.9021	0.8177	0.9969
✓	✓	SwinUNeTR	0.8625	0.9911	0.9037	0.8249	0.9969

**Table 7:** Ablation Studies on Contributions of Architecture and Modules on Brain Tumor MRI Testing Set When 40% of Training Set is Annotated. Checkmarks indicate that the corresponding component is included.

Model Consistency	Data Consistency	Network	Dice	Acc	Pre	Sen	Spe
✓		3D-UNet	0.8434	0.9901	0.8991	0.7943	0.9969
✓	✓	3D-UNet	0.8452	0.9902	0.9040	0.7936	0.9971
✓		UNETR	0.8680	0.9914	0.9009	0.8374	0.9968
✓	✓	UNETR	0.8657	0.9913	0.9056	0.8292	0.9970
✓		SwinUNeTR	0.8673	0.9914	0.9079	0.8302	0.9971
✓	✓	SwinUNeTR	0.8705	0.9916	0.9042	0.8392	0.9969

**Table 8:** Ablation Studies on Contributions of Architecture and Modules on Brain Tumor MRI Testing Set When 80% of Training Set is Annotated. Checkmarks indicate that the corresponding component is included.

Model Consistency	Data Consistency	Network	Dice	Acc	Pre	Sen	Spe
✓		3D-UNet	0.8424	0.9900	0.9020	0.7901	0.9970
✓	✓	3D-UNet	0.8516	0.9904	0.8912	0.8155	0.9965
✓		UNETR	0.8671	0.9913	0.8975	0.8387	0.9967
✓	✓	UNETR	0.8749	0.9918	0.9016	0.8498	0.9968
✓		SwinUNeTR	0.8766	0.9919	0.9036	0.8511	0.9968
✓	✓	SwinUNeTR	0.8783	0.9920	0.9008	0.8570	0.9968

**Table 9:** Complete ablation study on the BraTS 2019 testing set using Dice score. DC denotes data consistency, MC denotes model consistency, and AW denotes the auxiliary weighting parameter.

Setting	10%	20%	40%	80%
Supervised only	0.6486	0.7645	0.7987	0.8455
DC only	0.8245	0.8248	0.8548	0.8613
MC only	0.8463	0.8578	0.8673	0.8766
MC + DC	0.8493	0.8625	0.8705	0.8783
MC + DC + AW	0.8564	0.8794	0.8651	0.8859

## Data Availability

The BraTS 2019 dataset used in this study is available from the official BraTS challenge organizers subject to their data access policy.
